# Non-canonical pathways in the pathophysiology and therapeutics of bipolar disorder

**DOI:** 10.3389/fnins.2023.1228455

**Published:** 2023-08-01

**Authors:** Rodrigo Machado-Vieira, Alan C. Courtes, Carlos A. Zarate, Ioline D. Henter, Husseini K. Manji

**Affiliations:** ^1^Department of Psychiatry and Behavioral Sciences, University of Texas Health Science Center, Houston, TX, United States; ^2^Experimental Therapeutics and Pathophysiology Branch, National Institute of Mental Health, National Institutes of Health, Bethesda, MD, United States; ^3^Deparment of Psychiatry, University of Oxford, Oxford, United Kingdom

**Keywords:** bipolar disorder, mania, depression, treatment, targets, biomarkers, neurobiology

## Abstract

Bipolar disorder (BD) is characterized by extreme mood swings ranging from manic/hypomanic to depressive episodes. The severity, duration, and frequency of these episodes can vary widely between individuals, significantly impacting quality of life. Individuals with BD spend almost half their lives experiencing mood symptoms, especially depression, as well as associated clinical dimensions such as anhedonia, fatigue, suicidality, anxiety, and neurovegetative symptoms. Persistent mood symptoms have been associated with premature mortality, accelerated aging, and elevated prevalence of treatment-resistant depression. Recent efforts have expanded our understanding of the neurobiology of BD and the downstream targets that may help track clinical outcomes and drug development. However, as a polygenic disorder, the neurobiology of BD is complex and involves biological changes in several organelles and downstream targets (pre-, post-, and extra-synaptic), including mitochondrial dysfunction, oxidative stress, altered monoaminergic and glutamatergic systems, lower neurotrophic factor levels, and changes in immune-inflammatory systems. The field has thus moved toward identifying more precise neurobiological targets that, in turn, may help develop personalized approaches and more reliable biomarkers for treatment prediction. Diverse pharmacological and non-pharmacological approaches targeting neurobiological pathways other than neurotransmission have also been tested in mood disorders. This article reviews different neurobiological targets and pathophysiological findings in non-canonical pathways in BD that may offer opportunities to support drug development and identify new, clinically relevant biological mechanisms. These include: neuroinflammation; mitochondrial function; calcium channels; oxidative stress; the glycogen synthase kinase-3 (GSK3) pathway; protein kinase C (PKC); brain-derived neurotrophic factor (BDNF); histone deacetylase (HDAC); and the purinergic signaling pathway.

## 1. Introduction

Bipolar disorder (BD) is highly prevalent. For instance, one meta-analysis of 25 studies found a pooled lifetime prevalence of 1.06 and 1.57% for BD type I and type II, respectively (Clemente et al., 2015). BD type I is characterized by manic episodes and at least one major depressive episode in the individual’s lifetime, while BD type II is diagnosed when a history of both major depressive episode and hypomanic episode are present (Bauer, 2022). Individuals with BD are symptomatic about half of the time, with depressive symptoms occurring three times as frequently as manic and hypomanic symptoms (Kupka et al., 2007). BD is also associated with high direct and indirect costs, high rates of comorbidity, increased risk of mortality, and higher suicide rates than the general population (Miller et al., 2014). Presently, few pharmacological therapies for either acute or maintenance treatment are available to treat the wide spectrum of symptom presentations associated with BD, contributing to issues with the implementation of evidence-based medicine for clinical care (Gomes et al., 2022). Individuals with BD are therefore often prescribed a combination of therapies in order to improve functional outcomes, with the ultimate goal of reducing the frequency, duration, and severity of episodes and increasing the amount of time spent in remission (Dean et al., 2018). While mood stabilizers like lithium remain the most common maintenance treatment, along with second-generation antipsychotics, treatment resistance—loosely defined as suboptimal treatment response or failure to respond to an adequate treatment trial—remains common (Dodd et al., 2015). As a result, individuals with BD tend to experience frequent relapses, significant impairment, and disability (Woo et al., 2020). In addition, experiencing multiple mood episodes has been linked to more severe course of illness, including treatment resistance, significant morbidity, increased suicidality rates, and worse long-term prognosis (Peters et al., 2016). In this context, a key path to discovering potential new therapies for BD is to conduct well-designed experimental therapeutic trials with medications that have emerged from classic neuropharmacology. These agents may have a novel mechanism of action and are often first approved by the FDA for other indications (Haggarty et al., 2020).

Understanding the neurobiology of BD is crucial for developing more effective treatments—whether based on novel or repurposed agents—and recent work in the field has thus focused on identifying relevant biomarkers. Such biomarkers have been identified for genetic variations, oxidative stress, inflammation, and neurotrophic factors, among others (Rowland et al., 2018), and some have shown promise for monitoring course of illness, tracking treatment outcomes, and informing clinical decision-making. Understanding the genetics of BD could also help find new pathways for therapies (Hou et al., 2016). Given the heterogeneous nature of BD’s phenomenology, illness trajectory, and response to treatment, identifying clinically meaningful subgroups with differential responses to specific treatments is a research priority (McIntyre et al., 2022).

**Figure 1:**
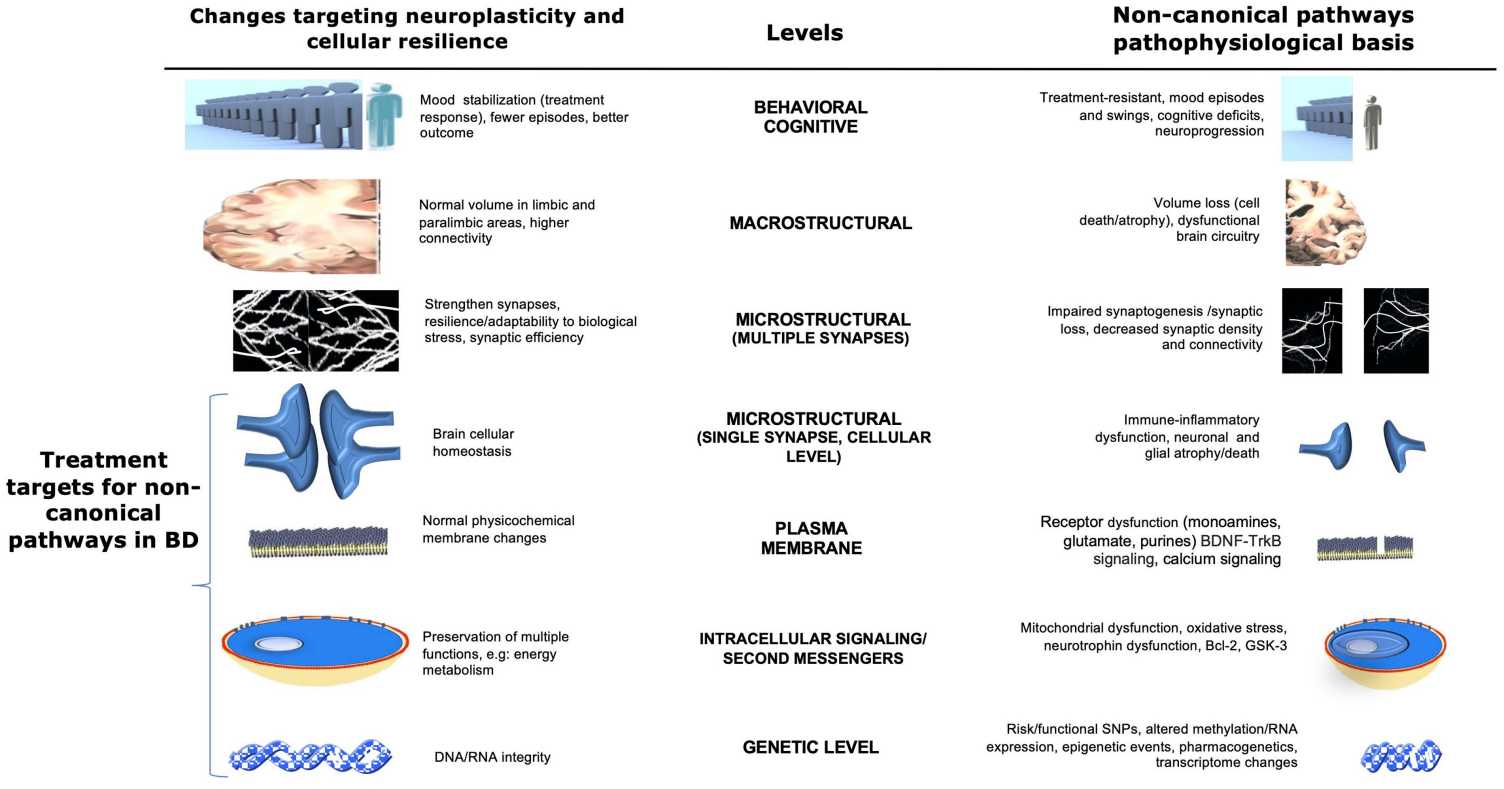
Potential non-canonical pathophysiological basis and therapeutic targets in bipolar disorder at multiple levels. This figure illustrates multiple non-canonical pathways and possible biological CNS targets for the development of new therapies for bipolar disorder. Areas of interest involve changes that affect the neuroplasticity and pathophysiological basis of the disease at several levels, from the behavioral through the CNS macro/microstructural and genetic levels. Changes in brain cellular homeostasis, membranes, energy metabolism, and DNA/RNA integrity (highlighted at the bottom left) represent direct targets for the development of new therapies involving non-canonical pathways. Adapted with permission from Machado-Vieira et al. (2014). BDNF, brain-derived neurotrophic factor; Bcl-2, B-cell lymphoma 2; ER, endoplasmic reticulum; GSK3, glycogen synthase kinase 3; SNP, single nucleotide polymorphism; TRK-B, tropomyosin receptor kinase B.

This review focuses on the role of non-canonical pathways in the pathophysiology of and therapeutics for BD. “Non-canonical” pathways are loosely defined as systems and pathways whose role in the disease course of BD remain unclear or unknown at present, in contrast to agents for BD that target classic or well-established pathways. Studying these new pathways and biomarkers may yield insights with broad clinical applications for individuals with BD. The article reviews several key topics, including: (1) the role of neuroinflammation as a significant factor in the pathophysiology of BD; (2) abnormalities in mitochondrial function and the potential for mitochondrial modulators to serve as effective treatment targets; (3) the link between calcium channels and BD; (4) the relationship between oxidative stress and BD; (5) the role of the glycogen synthase kinase-3 (GSK3) pathway, which is implicated in the mechanism of action of many drugs used to treat BD; (6) the role of protein kinase C (PKC), an intracellular signaling cascade with potential therapeutic relevance; (7) the role of neurotrophic factors, specifically the relationship between brain-derived neurotrophic factor (BDNF) and mood disorders; (8) the role of histone deacetylase (HDAC), an epigenetic marker; and (9) the purinergic signaling pathway and its neuromodulatory effects in the pathophysiology of BD. Each of these areas is reviewed in a framework that explores multiple biological levels (see Figure 1) and discussed in the context of both understanding the mechanisms underlying BD and developing safe and effective treatments for this debilitating condition.

## 2. The immune-inflammatory system

Inflammation is a non-canonical pathway that has received much attention in recent years. Studies have shown that chronic inflammation can be triggered by a variety of factors, including stress, infection, and poor diet, all of which can contribute to the development of mood disorders, including BD [reviewed in Yuan et al. (2019)]. The underlying mechanism is thought to be the ability of inflammation to alter neurotransmitter function and disrupt neuroplasticity, leading to changes in mood and behavior.

**Figure 2:**
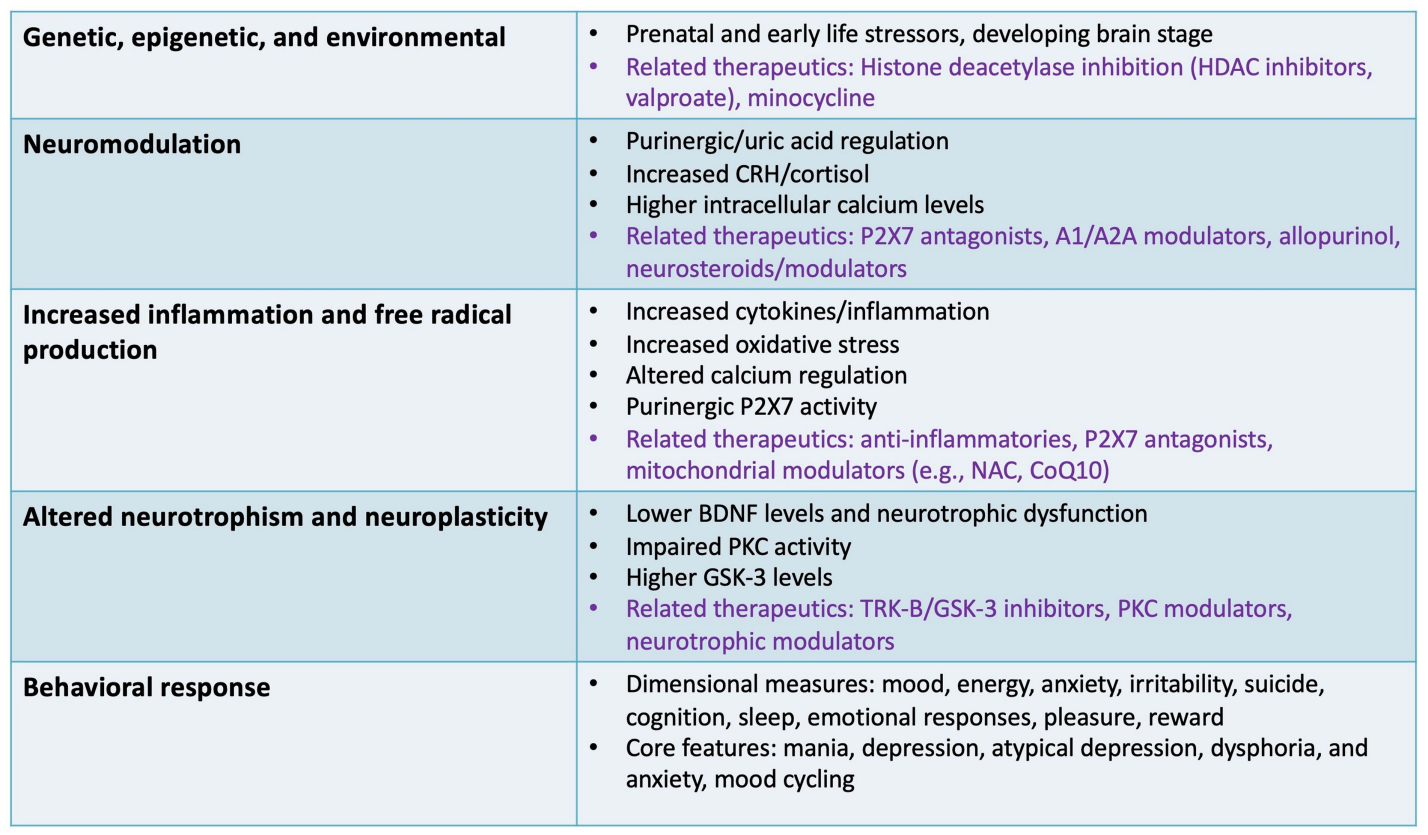
Specific targets and outcomes involving non-canonical pathways. This figure illustrates several non-canonical pathways involved in the pathophysiology of bipolar disorder that may be relevant to drug development, including inflammatory, neurotrophic, epigenetic, and neuromodulatory targets. Specific therapeutics (in purple font) are highlighted for some of the specific pathways discussed as potential non-canonical therapeutic targets and specific agents/classes under development. A1/A2A, adenosine receptors; BDNF, brain-derived neurotrophic factor; CRH, corticotropin-releasing hormone; CoQ10, coenzyme Q10; GSK-3, glycogen synthase kinase 3; HDAC, histone deacetylase; NAC, N-acetylcysteine; P2X7, P2X purinoceptor 7; PKC, protein kinase C; TRK-B, tropomyosin receptor kinase B.

Immune-inflammatory signaling is partially mediated by circulating peripheral blood mononuclear cells (PBMCs), including lymphocytes, leukocytes, and neutrophils, as well as central microglia and astrocytes. It involves an array of interacting signaling molecules (McNamara and Lotrich, 2012). There is a strong connection between inflammatory signaling and cellular stress, neuronal viability, and overt symptomatology (Jones et al., 2021). In addition, the relationship between inflammatory markers and brain structure in individuals with BD is well-known, both related to peripheral and central nervous system (CNS) inflammation (Fries et al., 2019). Individuals with mood disorders have been shown to have higher levels of pro-inflammatory cytokines and acute-phase proteins than healthy volunteers (Figure 2). The most consistent findings implicate interleukin 1 (IL-1) beta, IL-2, tumor necrosis factor-alpha (TNF-alpha), C-reactive protein (CRP), and IL-6, which increases Th1 cell functioning [reviewed in Solmi et al. (2021)]. For instance, studies found increased IL-1 beta in cerebrospinal fluid from individuals with BD and, in postmortem studies, higher levels of nuclear factor-kB (NF-kB), IL-1 receptor, glial fibrillary acidic protein (GFAP), and mRNA levels of IL-1 beta in the brains of individuals with BD compared to healthy volunteers (Rao et al., 2010). Higher peripheral concentrations of CRP have also been observed in individuals with BD compared to healthy volunteers, and these levels have been shown to decrease when symptoms remit (Fernandes et al., 2016). Another study similarly found that baseline CRP levels were significantly higher in individuals with BD than in healthy volunteers, and that individuals with BD treated with adjunctive cyclooxygenase-2 (COX-2) inhibitors had significantly lower CRP levels than those who received placebo (Edberg et al., 2018). Finally, another study found a direct correlation between cortical thickness in the right anterior cingulate cortex and chemokine ligand-2, IL-8, and TNF-alpha levels in bipolar depression. The authors suggested that these findings could be related to increased intracellular and extracellular water (swelling) (Poletti et al., 2019). Collectively, the evidence suggests that the deleterious effects induced by these cytokines can activate inflammatory pathways and alter energy homeostasis, synaptic neurotransmission, and oxidative balance.

Inflammation may also activate the kynurenine (KYN) pathway. Notably, KYN metabolites act as immune system modulators and can be either neuroprotective [e.g., kynurenic acid (KA)] or neurotoxic [e.g., quinolinic acid (QA)]. Several large meta-analyses found that alterations in KYN pathway metabolites were associated with mood, psychotic symptoms, and cognitive function in individuals with BD. Specifically, one large meta-analysis found that levels of KYN, KA, tryptophan (TRP), and the KA to quinolinic acid (QA) ratio were decreased in individuals with BD (Marx et al., 2021). Another meta-analysis of 16 studies found that individuals with BD had lower levels of TRP, KYN, KA, and xanthurenic acid as well as lower KA to KYN and KA to QA ratios than healthy volunteers (Bartoli et al., 2021b). That study also found that individuals experiencing a manic episode had the greatest reductions in TRP levels, and that individuals experiencing a depressive episode had the greatest reductions in KA levels. However, some of this evidence has been mixed. For instance, a more recent review found that some features of BD, such as psychotic episodes and suicide attempts, may also be associated with an imbalance in kynurenine pathway metabolism, though this finding requires replication (Bartoli et al., 2022). In addition, another recent meta-analysis of eight CSF studies found no evidence of increased KA levels or differences in TRP levels associated with BD (Inam et al., 2023). Intriguingly, a recent study found that serum KYN levels were lower in BD patients than in healthy volunteers, but that hospitalized individuals with BD had lower KYN levels than stabilized outpatients; that study also found that longer duration of illness was associated with altered levels of TRP, KYN, QA, xanthurenic acid, and picolinic acid, suggesting that KYN pathway metabolites may serve as biomarkers related to acute symptomatology and longer duration of illness (Skorobogatov et al., 2023).

Additional support for a pro-inflammatory hypothesis in BD comes from evidence that antipsychotics and lithium both downregulate the expression of inflammatory genes in the monocytes of individuals with BD. Two potential mechanisms have been identified as key mediators of this link between immune-inflammatory signaling and mood dysregulation: altered central serotonin metabolism and hypothalamic–pituitary–adrenal (HPA) axis dysregulation (McNamara and Lotrich, 2012). In this context, growing evidence suggests that mood disorders are linked to increased immune-inflammatory signaling that contributes to cognitive dysfunction (Huang et al., 2022); one review study noted that this increased signaling, in turn, can contribute to disturbances in central serotonin neurotransmission and HPA axis reactivity and damage to white and gray matter (Rosenblat and McIntyre, 2017). Preclinical studies have also shown that inducing immune-inflammatory signaling in animals can lead to depressive behaviors and neurochemical changes like those seen in human mood disorders (Drevets et al., 2008). However, further research is needed to fully understand these mechanisms and identify potential therapeutic targets. Nevertheless, because mood stabilizers and atypical antipsychotics suppress common components of the immune-inflammatory signaling pathway, researchers have hypothesized that adjunctive use of anti-inflammatory agents might enhance the efficacy of medications currently used to treat BD.

Building on this hypothesis, clinical trials are underway to evaluate the impact of various anti-inflammatory agents, such as non-steroidal anti-inflammatory drugs (NSAIDs), aspirin, minocycline, and infliximab in mood disorders (Fitton et al., 2022). For example, randomized clinical trials demonstrated the potential of minocycline, a tetracyclic antibiotic with antioxidant, radical scavenging, anti-inflammatory, and antioxidant characteristics to treat bipolar depression (Kraus et al., 2005; Garrido-Mesa et al., 2013; Savitz et al., 2018). In contrast, another randomized, controlled trial of adjunctive minocycline and celecoxib to treat bipolar depression in a 2 × 2 factorial design found no significant differences between the groups (minocycline plus celecoxib, minocycline plus placebo, placebo plus celecoxib, and placebo plus placebo) (Husain et al., 2020). A randomized, controlled trial with adjunctive infliximab, a monoclonal antibody, found no superiority over placebo for the primary outcome, though a secondary analysis identified a potential benefit among a subgroup with a history of childhood maltreatment (McIntyre et al., 2019).

Notably, a meta-analysis that examined the effects of anti-inflammatory agents as adjunctive therapy for BD found insufficient evidence to recommend the clinical use of any such agent, including NSAIDs, omega-3 fatty acids, or pioglitazone (Rosenblat et al., 2016). Only adjunctive N-acetylcysteine (NAC) was found to have a statistically significant antidepressant effect, but this was based on a single study and not a pooled sample (Berk et al., 2019). However, it should be noted that most studies in the meta-analysis randomized participants during an acute depressive episode, though some did not specify the phase of illness or stated that participants had experienced a recent mood episode within the past 6 months to 1 year. It should also be noted that while the meta-analysis found no significant evidence for the clinical use of any anti-inflammatory agent as adjunctive therapy for BD, measuring relapse rates and change in depression severity rating scale scores in response to adjunctive anti-inflammatory agents versus conventional therapy alone would still be of interest from a relapse prevention perspective. In addition, future studies should consider measuring changes in depression severity scores for currently depressed versus euthymic participants and focusing on specific clinical domains, such as anhedonia, to better understand how anti-inflammatory therapeutics may impact specific, clinically-relevant symptoms.

Another potential therapeutic pathway under investigation is the NLR family pyrin domain containing 3 (*NLRP3*) gene, which is hypothesized to be a central mediator of inflammation that links other cellular stress systems to immune cell activation (Figure 2). Treatment with the tricyclic antidepressant amitriptyline reduced levels of NLRP3 and caspase-1 in peripheral blood mononuclear cells as well as serum IL-beta and IL-18 levels in individuals with major depressive disorder (MDD) (Alcocer-Gómez et al., 2014). Other agents with the potential to modulate the immune system, such as probiotics and multipotent mesenchymal stromal cells, are also under investigation for bipolar depression (Gallagher et al., 2019; Aslam et al., 2020). Another proposed target for pharmacotherapy is the arachidonic acid cascade (McNamara and Lotrich, 2012). Animal studies found that lithium, valproate, and carbamazepine all decreased arachidonic acid turnover, although docosahexaenoic acid did not (Rao et al., 2008). This decrease was thought to be mediated by the calcium-dependent cytosolic isoform of the enzyme phospholipase A2 (PLA2) in rat brain phospholipids, suggesting that the therapeutic actions of mood stabilizers are at least partly mediated by COX-2 substrate sequestration in phospholipids and associated reductions in prostaglandin-E2 production.

Another review article also sought to explore the possible connection between the enteric nervous system and the central nervous system (CNS), otherwise known as the “microbiome-gut-brain axis” (Carabotti et al., 2015); this latter term refers to a complex, bidirectional communication system that modulates both bowel and behavioral function. An imbalance in gut microbiota is thought to drive the chronic inflammation associated with BD that is known to increase risk for both neuroprogression and medical comorbidities (Vaccariello and Nguyen, 2022). Microbiome research in psychiatry is in its relative infancy, but there has been a long-standing association between gastrointestinal pathologies and psychiatric disorders (Lee et al., 2015; Severance et al., 2015). Intriguingly, recent research suggests that an imbalance in gut microflora (dysbiosis) may cause luminal inflammation, facilitating microbial translocation into systemic circulation (Dickerson et al., 2017).

Finally, it should be noted that a recent review article identified inflammasome activation as an inflammatory signaling mechanism in BD (Jones et al., 2021). The same review analyzed the common thread between all inflammatory signaling pathways of the disease process and concluded that the heterogeneity and lack of objective markers make progress in this area of research particularly complicated. To move the field forward, it may be necessary to re-assess conventional approaches due to the individual variation in the expression of pathophysiological mechanisms.

## 3. Mitochondrial function

Mitochondria are the leading energy factories of eukaryotic cells. The brain, in particular, depends on mitochondrial activity because of its high energy use levels and inability to store large amounts of energy reserves in the form of glycogen (Allen et al., 2018). The primary role of mitochondria is to convert the products of carbohydrate, protein, and fat metabolism to CO_2_ and water using critical enzymes of the electron transport chain. Dysfunction in these mitochondrial complexes is believed to play an essential role in the pathogenesis of some chronic diseases (Johannsen and Ravussin, 2009), including BD, and primary mitochondrial disease is known to increase susceptibility to BD (Colasanti et al., 2020). For instance, one study found that mitochondria unique deletions and copy number were both increased, while complex I activity was decreased in the postmortem human dorsolateral prefrontal cortex (DLPFC) of individuals with BD compared to healthy volunteers (Das et al., 2022).

The putative link between BD and mitochondrial dysfunction rests on the notion that mitochondrial dysfunction is a critical pathological factor that can be intimately linked to a wide range of processes associated with treatment outcomes and disease progression or severity (Scaini et al., 2016, 2021). A recent preclinical study of low-dose rotenone-induced manic- and depressive-like behaviors corroborated the concept that BD involves a gradual decrease in mitochondrial function, with symptoms beginning only when a threshold is reached or when an event occurs that requires fully functional cells, making the individual more vulnerable to environmental factors that target mitochondrial function (Damri et al., 2023). Mitochondria also critically regulate energy production and control the production of reactive oxygen species (ROS), which are also involved in mood disorders (Barnham et al., 2004). Mitochondria also control calcium regulation, which is thought to play a key role in the pathophysiology of mood disorders (see Section 4.0, below) based on evidence that individuals with BD have elevated calcium levels in peripheral cells that can be reversed by administration of antidepressants and mood stabilizers (Harrison et al., 2018). In addition, N-acetyl-aspartate (NAA), which is produced in mitochondria, is one of the most abundant brain metabolites and serves as an important marker of neuronal viability. Recent magnetic resonance spectroscopy (MRS) studies found decreased NAA levels in individuals with BD compared to healthy volunteers (Kubo et al., 2017; Rosso et al., 2017). Another study found significant increases in NAA levels in individuals with BD after 12 weeks of treatment with the mood stabilizer and anticonvulsant lamotrigine (Croarkin et al., 2015). BD has also been associated with mutations and polymorphisms in mitochondrial DNA, a critical finding given the high heritability and significant genetic correlation of BD (Munakata et al., 2007; Soeiro-de-Souza et al., 2013; Chang et al., 2014; Kasahara and Kato, 2018; Wang et al., 2018; Schulmann et al., 2019).

One key study found altered metabolic activity in the brain that led to a shift toward lactic acidosis during mood episodes (Machado-Vieira et al., 2017b). Increased cingulate cortex lactate levels were observed in participants with BD experiencing a depressive episode compared to matched healthy volunteers, reinforcing that lactate levels may be a state biomarker in BD. The same study found that 6 weeks of treatment with lithium monotherapy significantly decreased cingulate cortex lactate levels. Another randomized, controlled trial of adjunctive CoQ10, a mitochondrial modulator, found that it improved depressive symptoms in participants with BD compared with placebo (Mehrpooya et al., 2018). In addition, a small, six-week, randomized, controlled trial found that treatment with the mitochondrial modulator creatine monohydrate led to higher remission rates of depressive symptoms (<12 in the MADRS) than those observed in the placebo group (52.9% vs. 11.1%, respectively), suggesting that creatinine may play an important role in treating depressive episodes in BD (Toniolo et al., 2018).

Collectively, the abnormalities in mitochondrial function associated with BD suggest fruitful avenues for improving our understanding of the pathophysiology of BD and identifying targets for mood stabilization (Das et al., 2022). In particular, studies exploring potential interventions that target direct mitochondrial function suggest that approaches targeting this organelle may improve clinical outcomes in BD.

## 4. Calcium channels

Calcium signaling is tightly regulated in various cellular processes, maintaining a delicate balance of intracellular concentrations through sequestration, buffering, and mobilization. These processes include neuronal excitability, neurotransmitter synthesis/release, synaptogenesis, and plasticity with mitochondria and endoplasmic reticula (ER) (Kato, 2019). Small perturbations in the balance between intracellular and extracellular calcium can trigger cell-death programs unresponsive to molecular rescue (Jones et al., 2020).

BD is characterized by altered intracellular calcium homeostasis. Indeed, altered intracellular calcium is considered the most reproducible cellular abnormality and biomarker in BD research (Zanetti et al., 2015), based primarily on *ex vivo* studies in cells taken from individuals with BD as well as healthy volunteers. Measures of intracellular calcium signaling are also increased in BD, with abnormalities appearing in both manic and depressive episodes (Harrison et al., 2018). In addition, genomic studies found that slow-gated L-type channels (LTCC) subunit-encoding genes, especially *CACNA1C*, are associated with BD, schizophrenia, and MDD (Craddock and Sklar, 2013; Heyes et al., 2015; Harrison, 2016). Lower cortical thickness in parietal and frontal cortices was also described in individuals with BD carrying the *CACNA1C* risk allele (Smedler et al., 2019); furthermore, a clinical study found that altered *CACNA1C* expression significantly affected the intrinsic spontaneous calcium activity of neural progenitors that play a crucial role in brain development, suggesting that *CACNA1C* may act as a molecular switch that increases susceptibility to psychiatric disease (Smedler et al., 2022).

Recent advances in our understanding of the molecular mechanisms that regulate intracellular calcium signaling have highlighted the role of intracellular calcium homeostasis in psychiatric disorders (Smedler et al., 2022). Researchers have used cellular models that minimize or eliminate confounding factors to better understand the abnormalities involved. This approach has yielded important observations suggesting that chronic treatment with the mood stabilizers lithium and valproate may have neuroprotective effects mediated through the normalization of ER stress response, stabilization of mitochondrial energetics, and attenuation of excitotoxic N-methyl-D-aspartate (NMDA)-mediated calcium influx (Warsh et al., 2004). These processes are critical for regulating intracellular calcium homeostasis. In particular, elevated basal levels and increased calcium release are associated with single nucleotide polymorphisms (SNPs) of *BCL-2*, with a direct relationship to developing BD (Machado-Vieira et al., 2011b).

Altered membrane excitability may disrupt several neural components responsible for controlling mood and behavior. In turn, modulating calcium signaling has multiple benefits, such as inducing long-term potentiation of synaptogenesis and acutely stabilizing neurotransmission (Kawamoto et al., 2012). The mood stabilizer lithium depletes inositol, a key calcium-signaling intermediary. For instance, lithium inhibits inositol-monophosphatase (IMPase) involved in phosphatidylinositol 4, 5-bisphosphate (PIP2) turnover, reducing calcium entry into the cell and thus overall neuronal excitability, explaining its ability to alter neurotransmission in BD (Berridge, 2014). Lithium also significantly reduces NMDA in receptor-stimulated calcium responses in animal and human models (Harrison, 2016). In addition, researchers found that induced stem-cell-derived (iPSC) hippocampal neurons from individuals with BD displayed a characteristic, hyperexcitable calcium signaling phenotype that was selectively reversed by lithium *in vitro* only in neurons derived from individuals who responded clinically to lithium (Mertens et al., 2015). The fact that the integrity of intracellular calcium homeostasis is critical to cell survival, as well as the notion that lithium’s effects on calcium signaling might be mechanistically relevant to its neuroprotective actions, is now thought to be critical to the clinical effects of this agent (Manji et al., 2000).

Interestingly, clinical studies also examined three widely used antihypertensive medications with LTCC activity (verapamil, diltiazem, and nimodipine) for the treatment of BD. When verapamil was compared with lithium and placebo in the treatment of manic episodes, lithium and verapamil both had similar antimanic effects that were superior to placebo (Giannini et al., 1984). However, subsequent studies with all three antihypertensive medications failed to replicate the antimanic efficacy of these agents over placebo (Janicak et al., 1998; Keck et al., 2000).

Gabapentinoids are also attractive candidate molecules for the treatment of psychiatric conditions. These agents act primarily by inhibiting neuronal voltage-gated calcium channel (VGCC) currents by binding to the α2δ auxiliary subunit that regulates channel trafficking and function. Although genes encoding α2δ subunits have not thus far been associated with psychiatric phenotypes, other VGCC genes, particularly the aforementioned *CACNA1C*, show robust transdiagnostic associations with multiple disorders, including BD (Harrison et al., 2018). However, a recent systematic review and meta-analysis that assessed the efficacy, acceptability, and tolerability of gabapentin and pregabalin found no robust evidence for the efficacy of these agents in BD, as assessed via the primary outcome measures of manic and depressive symptoms evaluated via rating scale scores (Hong et al., 2022). Thus, despite the attractive genetic and pharmacological rationale for the use of gabapentinoids, further evidence of their efficacy and safety is required. Development of modified α2δ ligands with a more beneficial therapeutic profile that target subtypes or isoforms may also be warranted.

While the findings reviewed above are intriguing, the regulation of free intracellular calcium is a complex, multi-faceted process, and the abnormalities observed in BD could arise from various levels. Future studies should seek to delineate the specific regulatory sites at which the impairment occurs in mood disorders. This, in turn, would help develop targeted therapies that seek to correct the calcium imbalance associated with BD during mood episodes.

## 5. Oxidative stress

Oxygen is vital for life, and a complex system of checks and balances exists within the human body to utilize this essential element. The failure of antioxidant defenses to protect against free-radical generation damages cell membranes, with resulting dysfunction that might impact neurotransmitters involved in psychiatric diseases (Flatow et al., 2013). As noted above, mitochondria generate ROS; when out of balance with antioxidant substances, ROS may induce toxic effects and cell damage to a variety of cell types (Barnham et al., 2004). ROS contribute to diminished neuroplasticity and neurogenesis and, in mood disorders, increase apoptosis and neurodegeneration (Fornito et al., 2007).

Oxidative stress is characterized by increased free radicals, which attack proteins, DNA, and lipids. During mood episodes, different substances formed by the conversion of molecules increase, including thiobarbituric acid reactive substances (TBARS), superoxide dismutase (SOD), catalase (CAT), and neuron-specific enolase (NSE), which are produced by lipid peroxidation of the cellular bilayer. The organism compensates for this excess through the action of different blood antioxidants, such as uric acid (UA) and bilirubin, or by the action of buffering substances, like glutathione (GSH) and different antioxidant enzymes (Jiménez-Fernández et al., 2021). A recent meta-analysis found that TBARS levels were increased in individuals with mania, depression, and euthymia, and significantly higher levels were observed in individuals with mania versus euthymia, suggesting that TBARS is an oxidative stress trait marker of BD (Jiménez-Fernández et al., 2021). The same study found that antioxidants administered in conjunction with antidepressants were promising potential treatments for bipolar depression, though future studies are needed to confirm this finding. Another study comparing individuals with BD receiving lithium with unmedicated individuals with BD experiencing a manic episode found that TBARS, SOD, and CAT were increased in the unmedicated participants compared to those receiving lithium, suggesting that this agent exerts antioxidant effects during mania (Machado-Vieira et al., 2007). Another well-known agent in this research area is NAC, which is thought to cross the blood–brain barrier and penetrate the cell membrane, transporting cysteine into the astrocyte to produce GSH. GSH, in turn, is an essential anti-oxidative compound that scavenges free radicals, modulates mitochondrial function and glutamatergic neurotransmission, has anti-inflammatory properties, decreases apoptosis, and induces neurogenesis. However, a recent meta-analysis found that adding NAC to standard therapy did not statistically significantly improve any of the outcomes examined in individuals with BD-I or BD-II, including symptomatology, functionality, and quality of life (Pittas et al., 2021).

More broadly, many questions about the complex mechanisms of oxygen regulation remain unanswered. As the studies reviewed above underscore, different mechanisms appear to be involved in expressing different mood states, suggesting the need to study depressive and manic states in BD separately (Pittas et al., 2021). Furthermore, it is likely that evaluating patterns of multiple biomarkers of oxidative stress will be required to decipher the relationship between ROS and BD. Additional studies are needed to measure blood oxidative stress and immune/inflammatory parameters transdiagnostically to help establish disease-specific biomarkers. Specifically, exploring different blood sources could help stratify target biomarkers for risk, diagnosis, state, stage, prognosis, and treatment response. Most importantly, large, well-designed clinical trials are needed to assess the potential therapeutic efficacy of antioxidants in the treatment of BD.

## 6. Glycogen synthase kinase 3-beta

Another non-canonical pathway of interest in psychiatry is the GSK3/Wnt signaling pathway, which has been shown to play a role in developing and maintaining neuronal circuits in the brain and regulating adult neurogenesis. GSK3 is a versatile enzyme that orchestrates several intracellular pathways, integrating signals from neurotransmitters, neurotrophic factors, and hormones (Du et al., 2010; Bartzokis, 2012). GSK3 has nearly 50 substrates in different intracellular compartments. However, it is particularly active in the nucleus and mitochondria, acting as a modulator for several processes related to neuronal function, such as gene expression, neurogenesis, myelination, synaptic plasticity, energy production, inflammation, and neuronal death and survival, through the phosphorylation of other enzymes (Li and Jope, 2010; Bartzokis, 2012; Valvezan and Klein, 2012). The two main isoforms of GSK3–GSK3α and GSK3β–are expressed throughout the brain, are partially active in unstimulated cells, and are regulated predominantly in an inhibitory manner by similar signaling mechanisms. When active, GSK3 blocks myelination, progenitor cell proliferation, and neurogenesis (Li and Jope, 2010; Bartzokis, 2012; Valvezan and Klein, 2012).

The discovery in 1996 that lithium inhibits GSK3 activity raised the possibility of a link between GSK3, mood regulation, and BD. Animal models, *in vitro* investigations, and clinical studies all suggested that GSK3 activity correlates with mood regulation, though a direct causal or genetic relationship has not yet been established (Li and Jope, 2010). Studies have also shown that modulating the Wnt pathway can benefit mood and behavior, suggesting that this may also be a promising target for treating mood disorders (Valvezan and Klein, 2012), and dysregulation of the Wnt pathway has been specifically linked to BD (Muneer, 2017). In addition to lithium, valproate, antipsychotics, and antidepressants also inhibit GSK3; this suggests that GSK3 may contribute to the clinical efficacy of these agents by promoting myelination and neurogenesis (Bartzokis, 2012). Consistent with this hypothesis, a diffusion tensor imaging (DTI) study performed in individuals with BD found that long-term treatment with lithium and a variant of the GSK3β promoter gene was associated with reduced enzymatic activity related to increased measures of myelination in crucial white matter tracts (Benedetti et al., 2013).

Despite these intriguing links, much remains unknown regarding how GSK3, as a probable mediator of lithium’s effects, is capable of stabilizing mood. Attempts to elucidate how GSK3 contributes to lithium’s effects have focused chiefly on biological functions that are affected by GSK3 and lithium. This work has identified many receptor-mediated signals regulated by GSK3 (Chatterjee and Beaulieu, 2022), and many more are likely to be identified soon. Furthermore, while some consequences of GSK3 inhibition correlate with the natural outcomes of lithium treatment, this overlap has yet to be thoroughly characterized. It is also possible that lithium may not address causative mechanisms but, rather, simply remediate symptoms by overriding the effects of BD on brain function (Chatterjee and Beaulieu, 2022). This scenario may be the most probable, given that GSK3 enzymes are also inhibited by several other drugs used in psychiatry, including antipsychotics and ketamine (Jope and Roh, 2006). Indeed, GSK3’s apparent non-pharmacological selectivity (Chatterjee and Beaulieu, 2022) and the associated risks for serious adverse effects (though these have yet to be proven) have, to date, impaired the development of better approaches for treating BD and other psychiatric disorders.

## 7. Protein kinase C

PKC signaling plays a crucial role in several pathological mechanisms, including neuronal excitability, neurotransmitter release, energy homeostasis, synaptic neurotransmission, glutamate signaling, neuroinflammation, neuroplasticity, mitochondrial dysfunction, oxidative stress, and apoptosis. At presynaptic terminals, high levels of PKC regulate neuronal excitability, neurotransmitter release, and neuroplasticity (Zarate et al., 2003, 2006; Zarate and Manji, 2009a; Chu et al., 2014; Jun et al., 2014; Pahl et al., 2014; Nam et al., 2015).

PKC is part of the family of calcium and phospholipid-dependent enzymes involved in mood regulation. The activation of conventional PKC (cPKC) isoforms (α, βI, βII, γ) requires calcium and diacylglycerol (DAG), whereas novel PKC isoforms (δ, ε, η, θ, μ) require DAG for activation (Zarate and Manji, 2009b). In the CNS, cPKC isoforms are the most common and highly expressed in mood regulation structures, such as the prefrontal cortex (PFC), amygdala, and hippocampus (Naik et al., 2000). One recent study found brain region-specific (PFC and temporal cortex) abnormalities in PKC function in BD, as evidenced by decreased PKC activity (Pandey et al., 2020). These abnormalities were related to decreased levels of the PKCα, PKCβI, PKCβII, and PKCε isoforms. The same study found no abnormalities in PKC function or in PKC isozyme expression in other brain areas of BD participants, strongly suggesting region-specific alterations of PKC in the pathophysiology of BD.

With the development of animal models of mania in the past few decades, the involvement of PKC in the manic phenotype has become more apparent. One key study found that PKCε activity plays a significant role in hyperlocomotion and oxidative stress in the methamphetamine (m-AMPH)-induced animal model of mania (Valvassori et al., 2020). The same study found that inhibition of PKCε activity by intracerebroventricular microinfusion of iPKCε prevented behavioral and oxidative disturbances associated with the mania model, suggesting that PKCε may act as a potential mediator in the pathophysiology of BD. In rats, PKC inhibition reduced manic-like behaviors and hippocampal cell degeneration in a sleep deprivation model of mania, whereas PKC activation induced manic and depressive behaviors (Abrial et al., 2013). The rats exposed to long-term PKC inhibition displayed depressive-like behaviors and decreased hippocampal cell proliferation, supporting the hypothesis that PKC plays a critical role in mood regulation. Finally, the same study found that quercetin, a non-specific PKC inhibitor, prevented methylphenidate-induced hyperlocomotion and lipid peroxidation in mice.

Evidence linking PKC and BD also comes from studies showing that the mood stabilizers lithium and valproate, which have been used for decades to treat BD, are PKC inhibitors. These agents inhibit PKC activity *in vitro* and *in vivo*, and chronic lithium treatment has also been shown to decrease PKC levels in the platelets of individuals with BD (Chen et al., 2000; Soares et al., 2000). In addition, tamoxifen, a drug approved to prevent and treat breast cancer, is a CNS-penetrant PKC inhibitor with demonstrated efficacy in acute manic or mixed episodes of BD, both adjunctively and as monotherapy (Zarate et al., 2007; Yildiz et al., 2008; Saxena et al., 2017). Second-generation antipsychotics, which are PKC inhibitors, are also effective for treating manic episodes in BD (McIntyre et al., 2020).

Interestingly, a recent randomized, controlled trial investigated endoxifen for the treatment of BD (Ahmad et al., 2021). This agent, which is a metabolite of tamoxifen, crosses the blood–brain barrier and is a direct PKC inhibitor. Endoxifen was found to be safe and effective for improving symptoms in individuals with BD-I who presented with acute manic episodes, with or without mixed features. Specifically, total Young Mania Rating Scale (YMRS) scores, disease severity as assessed by Clinical Global Impressions-Bipolar Version (CGI-BP) and CGI-Severity (CGI-S) scale scores, and treatment response and remission rates were all improved. Time to remission was observed as early as Days 4 with endoxifen versus Days 7 with valproate, though the two interventions had similar response and remission rates at the study endpoint of Day 21. This earlier time to remission in acute mania suggests a somewhat faster mode of action that might be due to endoxifen’s direct PKC inhibition (Ahmad et al., 2021).

Finally, a meta-analysis of 8,700 individuals with either MDD or BD found that suicidality was significantly associated with the genetic locus for PKCε (Perlis et al., 2010). Another study that reviewed preclinical and clinical studies found that PKC levels and membrane-bound PKC activity were increased in cortical homogenates of individuals with BD compared to healthy volunteers (Saxena et al., 2017). In addition to corroborating hyperactive PKC signaling in BD, this finding suggests that studying downstream targets of PKC in BD may yield additional therapeutic targets. More research with PKC inhibitors is needed to develop safe and specific therapies for BD based on validated clinical studies.

## 8. Neurotrophins

Brain-derived neurotrophic factor (BDNF) plays a critical role in synaptic plasticity and memory. The hippocampal expression of BDNF decreases in response to chronic stress (Figure 2), and changes in BDNF expression have been strongly associated with both normal and pathological aging, particularly in structures essential for memory processes and psychiatric diseases (Miranda et al., 2019). Many studies have confirmed the strong relationship between BDNF and mood disorders in general and BD in particular [reviewed in Lin and Huang, (2020)].

The mood stabilizer lithium has relevant neurotrophic and neuroprotective effects *in vitro* and *ex vivo*. One preclinical study treated rat cerebral cortical neurons with lithium and found enhanced BDNF expression (Hashimoto et al., 2002). The same study found that the neuroprotective effects of lithium were absent in neurons in *Bdnf* knockout mice, suggesting that BDNF may play a role in lithium’s mechanism of action. From a clinical perspective, lithium—the most effective medication for relapse prevention in BD—exerts neuroprotective effects on the CNS and increases BDNF expression in animal and human models (Rybakowski, 2014; Malhi and Outhred, 2016). Other clinical studies found that lithium increased gray matter volumes in individuals with BD, an effect potentially mediated by its neurotrophic properties (Sassi et al., 2002).

It should be noted here that, like BDNF, vascular endothelial growth factor (VEGF) is associated with hippocampal neurogenesis (Fournier and Duman, 2012). However, despite evidence of increased VEGF levels in individuals with MDD compared with healthy volunteers, a meta-analysis found no differences in VEGF levels in individuals with BD (Pu et al., 2020). In addition, using a systems model of the human prefrontal transcriptional network, researchers highlighted the importance of early growth response gene 3 (*EGR3*), which is known to be modulated by BDNF (Pfaffenseller et al., 2016). One hypothesis suggests a positive feedback loop wherein BDNF signaling abnormalities lead to reduced *EGR3* expression, thus impairing neuroplasticity and resilience, increasing vulnerability to stress, and further lowering BDNF expression (Pfaffenseller et al., 2018).

From a pharmacological perspective, studies have examined neurotrophic agents not originally designed or used for mood disorders that might nevertheless improve cognition in individuals with mood disorders, including BD. Such agents include liraglutide, a glucagon-like peptide-1 receptor (GLP-1R) agonist with neurotrophic properties (Mansur et al., 2017; Park et al., 2018). One preclinical study found that liraglutide activated mammalian target of rapamycin complex 1 (mTORC1) signaling and α-amino-3-hydroxy-5-methylisoxazole-4-propionic acid (AMPA) receptors, increased the expression of BDNF and selected synaptic proteins, and enhanced neurite outgrowth and synaptic density under toxic conditions in rodents (Fries et al., 2019). A subsequent clinical study found that liraglutide was safe and well tolerated in non-diabetic individuals with mood disorders and that it had beneficial effects on cognitive function. These effects appeared to occur independently of liraglutide’s mood-related effects and might be moderated by metabolic status, suggesting a potential therapeutic target for individuals with BD who also suffer from cognitive decline (Mansur et al., 2017). Interestingly, vitamin D3 (VitD) has also been examined in this context. VitD crosses the blood–brain barrier, and its receptors are widely distributed in the CNS. Chronic VitD deficiency may accelerate the process of neuronal degeneration and cognition in rats (Bakhtiari-Dovvombaygi et al., 2021). Preclinically, prolonged exposure to unpredictable chronic mild stress (UCMS) resulted in decreased hippocampal expression of BDNF (Khairy and Attia, 2021), and treatment with VitD increased BDNF levels and reversed UCMS-related changes in BDNF concentrations in the hippocampus (Bakhtiari-Dovvombaygi et al., 2021; Khairy and Attia, 2021). However, a 12-week, double-blind, randomized, controlled study of VitD versus placebo supplementation found that VitD did not significantly improve the symptoms of bipolar depression (Marsh et al., 2017).

Most importantly, BDNF appears to be a beneficial tool for assessing disease activity in BD (Fernandes et al., 2014). Although mixed results have been observed, a large meta-analysis found decreased BDNF levels in manic and depressive episodes that correlated with episode severity and returned to normal in euthymia (Fernandes et al., 2015), suggesting that BDNF may act as a biomarker of both disease activity and disease stage. To assess whether pharmacological treatment of a mood episode induced changes in peripheral BDNF levels, two within-group meta-analyzes of longitudinal studies were conducted, one of BDNF changes before and after treatment of a manic episode and one of BDNF levels before and after treatment of a depressive episode. The six studies analyzed in this specific meta-analysis included 52 cross-sectional or longitudinal studies comprising 6,481 participants (Fernandes et al., 2015). BDNF levels were found to be moderately decreased in individuals with BD experiencing manic episodes and significantly decreased during depressive episodes. There was no association between BDNF levels and duration of illness in euthymia, suggesting that peripheral BDNF levels can serve as a biomarker for disease activity in BD, though the researchers noted that there were insufficient data to determine whether this biomarker reflected treatment response or prognosis (Fernandes et al., 2015). Direct comparisons across distinct mood states demonstrated that the reduction in peripheral BDNF levels was comparable in mania and depression and that both were equally reduced compared to euthymia. In addition, the same study found that peripheral BDNF levels increased after successful treatment of a manic episode but not of a depressive episode.

Taken together, the evidence reviewed above underscores that while the role of BDNF receptors in drug development for mood disorders failed to show promise, peripheral BDNF may be potentially important as a multi-use biomarker of disease activity. To further investigate the role of BDNF and other neurotrophins, BDNF levels will need to be compared at various timepoints across the course of illness. Nevertheless, the causal relationship between alterations in BDNF levels and changes in mood states in BD remains uncertain. For instance, it is unclear whether decreased peripheral BDNF levels precede the initiation of a mood episode or vice versa, whether increased peripheral BDNF levels precede recovery from a mood episode or vice versa, or whether changes in BDNF levels and symptoms co-occur. Before solid conclusions about this possible biomarker can be drawn, therapeutic target studies with improved designs and reduced noise and bias are necessary.

## 9. Histone deacetylase inhibition

Although mood disorders have not traditionally been regarded as developmental disorders, there has been a growing appreciation for the role of gene–environment interactions and early life events in their etiology. Epigenetics, in particular, is thought to mediate various environmental factors involved in the pathophysiology of major psychiatric disorders, including mood disorders (Rutten and Mill, 2009; Legrand et al., 2021). For instance, studies suggest that molecular brain adaptations may contribute to the pathophysiology of BD, and that early-life stressors may increase risk for the first episode of BD in susceptible individuals, perhaps due to deficits in producing more “plastic” chromatin (Machado-Vieira et al., 2011a). Increasing chromatin plasticity, in turn, may lead to stronger synaptic connections and long-term behavioral and mood regulation.

Currently, only preclinical studies have investigated HDACs as potential therapeutic targets for BD. HDAC inhibitors include hydroxamates, sodium butyrate (SB), and valproate. Interestingly, valproate was found to inhibit HDAC activity *in vitro* and may have neuroprotective effects (Göttlicher et al., 2001). In rodent models of depression associated with early life stressors, central infusion of HDAC inhibitors blocked histone deacetylation and induced antidepressant-like effects (Schroeder et al., 2007). Chromatin remodeling, including changes in histone acetylation, might thus play a role in the pathophysiology and treatment of mood disorders. In addition, some preclinical studies found that HDAC inhibitors could reverse mania-like behaviors and modulate epigenetics in animal models (Varela et al., 2020). A preclinical study that administered the HDAC inhibitor SB either alone or in conjunction with the selective serotonin reuptake inhibitor (SSRI) fluoxetine found that this agent exerted antidepressant-like effects in mice (Schroeder et al., 2007). Another preclinical study found that histone methylation was not reversed by treatment with antidepressants and that chronic imipramine induced long-lasting H3 hyperacetylation at the BDNF P3 and P4 promoters in the social defeat model of depression (Duclot and Kabbaj, 2015). Further investigation of the mechanism for this stable modification revealed that chronic imipramine downregulated HDAC5 in these mice, and that HDAC5 overexpression completely blocked the antidepressant efficacy of imipramine in the social defeat protocol. Taken together, these findings suggest that HDAC5 downregulation plays an essential role in the therapeutic actions of imipramine. Thus, other agents that inhibit HDAC5 inhibition—though not yet clinically available—may possess antidepressant effects (Tsankova et al., 2006). However, the synthesis of more specific HDAC inhibitors is still in its infancy, as is identifying nonhistone targets of these agents. Given chromatin’s activity and continuous adaptations, the therapeutic use of HDAC inhibitors to treat mood disorders is promising, though the half-life and affinity of any HDAC inhibitor may also define its potential risk for toxicity and undesirable side effects *in vivo* (Machado-Vieira et al., 2011a). Potential side effects must be considered, and appropriate dosing and confirmation of blood–brain barrier penetration remain necessary.

It should be noted that although direct evidence for epigenetic dysfunction in BD is still limited, advances in epigenomic profiling technologies offer an exciting opportunity to investigate molecular modifications to DNA and histones (Legrand et al., 2021). HDACs have several subtypes and biological targets, and a major concern with development of these agents is the risk of off-target unpredictable adverse effects, which has limited drug development in this area. Nevertheless, epigenomic profiling technologies may help identify the mechanisms by which specific HDAC subunits act upon complexes and downstream targets to bring about epigenetic changes in gene expression relevant to the therapeutics of mood disorders. Because the epigenetic machinery is dynamic and modifications can potentially be reversed, understanding these mechanisms is essential for identifying new prevention and therapeutic approaches.

## 10. The purinergic system

Purines are essential in energy metabolism, both intracellularly with ATP and in the extracellular space with adenosine and ATP as critical regulators of neurotransmission (Burnstock, 2006). Extracellular ATP acts on purinergic receptors P2X (P2XR) and P2Y (P2YR), activating several intracellular signaling cascades. ATP is metabolized into ADP, adenosine 5′-monophosphate (AMP), and adenosine by ectoenzymes that directly regulate extracellular purine concentrations (Gonçalves et al., 2022). While only limited clinical data are available, extensive basic and preclinical research supports the importance of adenosine and the purinergic system in the pathophysiology of BD. For instance, one key study discussed different purinergic system components and purinergic receptors according to their relevance to BD and found that the actions of allopurinol, UA, and the breakdown of ATP to adenosine were linked to many classic BD symptoms (Cheffer et al., 2018).

UA is an essential nitrogenous product of purinergic metabolism (ATP and adenosine). It modulates endogenous functions like sleep, appetite, cognition, memory, motor activity, and social interaction and is directly related to specific manic traits such as impulsivity, irritability, increased drive, disinhibition, and hyperthymia (Machado-Vieira et al., 2002; Lorenzi et al., 2010; Bartoli et al., 2017a). Evidence suggests that, while euthymic, plasma UA level abnormalities are absent in individuals with BD, supporting the notion that purinergic system dysfunction is critical in BD (Machado-Vieira et al., 2008, 2017a; Bowman et al., 2010; Albert et al., 2015; Muti et al., 2015). Furthermore, this finding suggests that UA may act as a state-dependent biomarker and outcome indicator in BD, underscoring the positive association between peripheral and central UA levels (Gubert et al., 2016). Relatedly, one study found elevated UA levels in drug-naïve participants experiencing their first manic episode (Salvadore et al., 2010). Another study that examined nationwide, population-based data found that individuals with BD were at increased risk of gout (Chung et al., 2010). Notably, higher serum UA levels have also been observed in individuals with BD experiencing an acute manic episode versus those experiencing a depressive episode or MDD participants, suggesting that UA levels may predict bipolar conversion in currently depressed individuals (Oliviera et al., 2019). In that study, inpatients with MDD who converted to BD had higher UA levels during their first depressive episode than individuals with MDD who did not progress to a diagnosis of BD. Identifying a diagnostic progression from MDD to BD would have substantial clinical implications, particularly in terms of influencing prognosis and treatment choice. In addition, elevated UA levels may be a trait marker for higher susceptibility to impulsivity, hyperthymic and irritable temperaments, and frank BD, particularly during manic episodes in drug-naive individuals. Thus, routine evaluation of serum UA levels when managing a depressive episode in the inpatient setting might help distinguish MDD from BD, and combining this laboratory finding with clinical factors may enhance prognosis (Oliviera et al., 2019).

Genetic studies revealed a specific SNP in the *P2X7* gene that encodes a CNS-expressed purine receptor associated with BD and MDD in animal models (Backlund et al., 2012). In humans, one study found that *P2X7* expression increased sleep deprivation and rapid cycling in individuals with BD, a finding with important implications for neuroinflammation and pathogenesis in BD (Masuch et al., 2016). As a result, targeted therapies for P2X7 in BD and MDD are currently being developed and tested. In animal models of mania—predominantly the amphetamine-induced hyperactive locomotion model—P2X7R antagonism was found to be a promising therapeutic candidate for BD (Gonçalves et al., 2022). Indeed, that study hypothesized that next-generation P2X7R antagonists are the most promising adjunctive therapy or monotherapy for BD and related disorders that affect the CNS. Critically, the diversity of metabolites and downstream interactions associated with this pathway underscores the significant potential for future therapeutic and biomarker development in this area, particularly P2X4R, A1R, A2AR, and P2Y1R (Bartoli et al., 2017c). In this context, it should be noted that several P2X antagonists are already being studied in clinical trials and may represent an important and innovative treatment target (Timmers et al., 2018; Koole et al., 2019; Recourt et al., 2020).

Another interesting area of research is adenosine receptor antagonism. These antagonists, such as caffeine and theophylline, exert anti-aggressive, anticonvulsant, and antipsychotic properties and can also enhance irritability, anxiety, and insomnia, suggesting that purinergic constituents independently contribute to overall disease process (Lara et al., 2006). A recent review of the potential role of adenosine receptors in BD underscored the role of the purinergic system and increased UA excretion in individuals with BD (Van Calker et al., 2019). Relatedly, allopurinol and its active metabolite oxypurinol are xanthine oxidase inhibitors. These agents exert their therapeutic effects by decreasing production of UA, superoxide, and hydrogen peroxide (Pacher et al., 2006). One study found that allopurinol increased CNS levels of adenosine and other purine metabolites when inhibiting xanthine oxidase and decreasing UA formation in newborn piglets (Marro et al., 2006). With regard to clinical studies, one key study found that allopurinol was effective in treatment-resistant mania associated with hyperuricemia (Machado-Vieira et al., 2001), and other trials subsequently demonstrated the efficacy of adjunctive allopurinol for improving manic symptoms (Akhondzadeh et al., 2006; Machado-Vieira et al., 2008; Jahangard et al., 2014). Meta-analyses of randomized, controlled trials also indicated that allopurinol had a small to moderate effect in BD (Hirota and Kishi, 2013; Bartoli et al., 2017b; Chen et al., 2018); however, the varying settings and mixed results of the individual studies suggest that caution is warranted when drawing conclusions about the usefulness of this agent (Lintunen et al., 2022). While these findings are promising, allopurinol as an add-on therapy for BD is still associated with significant challenges, including study design, heterogeneity of primary BD medication, and the doses and duration of its administration. Additional studies with allopurinol are needed to understand the potential of these therapies as well as the purinergic system’s role in BD.

## 11. Conclusions and future directions

Although several effective treatments are available for BD, symptoms persist for many individuals despite appropriate management. Indeed, the main impetus for investigating new therapies that target different pathways is that current treatments for BD are associated with delayed onset of action and significant side effects. As discussed above, the neurobiology of BD is complex and probably involves mitochondrial dysfunction, oxidative stress, decreased neurotrophic factors, and changes in immune-inflammatory systems (Vieta et al., 2018). All of these factors may not only be related to the etiology of BD but also be influenced by disease progression (Scaini et al., 2020). Improved clarification of the neurobiology of BD and the identification of new, clinically relevant targets is expected to provide insights about developing novel agents as well as repurposing drugs already approved for other indications to treat both mania and bipolar depression. In this context, a recent meta-analysis that examined drugs repurposed as adjunctive treatments for acute mood episodes concluded that this can be a fruitful approach (Bartoli et al., 2021a). Importantly, the non-canonical biological pathways reviewed above are biologically integrated, which may have diagnostic and therapeutic implications or might simply represent an epiphenomenon. As an example, inflammatory dysfunction in BD is also related to alterations in molecular, physiological, and brain microstructure (Berk et al., 2011).

Many of the agents reviewed above—drawn from a wide range of new options and pathways—are quite promising, including those related to the immune-inflammatory pathways, HDAC inhibitors, and P2X7R antagonists. Some of the agents described above, like calcium channel blockers, HPA-axis targeted treatments, and xanthine oxidase inhibitors, are well-known drugs used to treat other diseases. These potential new therapeutic targets have multiple systemic effects, from the genetic to the psychosocial. Significantly for the field, some of these interventions—like mitochondrial dysfunction and oxidative stress-targeted therapies, including a wide range of nutraceuticals—may ultimately help prevent or halt symptoms from their earliest stages when used adjunctively. Targeting different biomarkers may ultimately influence disease progression and even reverse or prevent some of the more severe disease presentations, for instance, preventing permanent macrostructural changes (e.g., reduced brain structures, irreversible inflammatory and metabolic changes; Figure 2).

As the field progresses, researchers and clinicians are focused on identifying more personalized and precise targets aimed at alleviating specific BD symptoms, which could significantly improve quality of life for patients. More broadly, the scientific community is already moving toward precision medicine for disease control and management, and BD is no exception. In this context, considering the similar biological characteristics of some diseases and the pleiotropic effects of drugs could benefit from computational methods (Brown and Patel, 2017; Jourdan et al., 2020). One such study compared genes in pathways associated with treatment-resistant depression to genes targeted by known drugs in order to identify candidate pharmacotherapies for repurposing. The results showed that the most often identified drugs included modulators of inflammation and immune systems, as well as agents targeting monoaminergic transmission, cell proliferation, and survival (Fabbri et al., 2021). In another study, blood gene expression biomarkers predicted depression in individuals with mood disorders, including BD, and compared them against new or repurposed candidate substances that target these biomarkers. The results suggested a possible pathway that could be used to identify biomarkers and stratify patient populations when preventing and treating BD, tools that could enable precision medicine for this condition (Le-Niculescu et al., 2021). Moving forward, researchers should focus on exploring well-designed translational approaches with robust and clinically oriented preclinical studies followed by randomized multi-center clinical trials with larger sample sizes. In addition, identifying brain circuits related to relevant clinical phenotypes can provide targets for neuromodulation and transdiagnostic predictive markers of treatment response, benefiting individuals with different psychiatric disorders, including BD.

Improving our understanding of the mechanism of action of drugs with antidepressant and mood stabilizing properties also continues to broaden the array of therapeutic possibilities. For example, explorations into the rapid-acting mechanism of action of ketamine, which has shown antidepressant efficacy in both MDD and BD, has improved our understanding of the pathophysiology of depression as well as stimulated the investigation of different glutamatergic agents to treat mood disorders, including bipolar depression (Das, 2020; Henter et al., 2021). With regard to biomarkers in particular, the work described above has contributed significantly to identifying state-, stage-, treatment response-, and prognosis-related biomarkers. Identifying additional biomarkers that would be more responsive to specific treatments as well as novel targets in non-canonical pathways will require a significant collaborative effort that encompasses preclinical and clinical studies, complex computational approaches, and appropriate study designs.

Nevertheless, developing novel and effective treatments in psychiatry is associated with several challenges, mainly related to the enormous complexity of the brain and its interaction with the environment. In addition, the heterogeneity of psychiatric diagnoses—as categorized via current classification systems—may have hampered advances in psychiatric treatment despite contributing to diagnostic reliability in psychiatry. This is because subgroups of patients with different patterns of neurobiological dysfunction may display distinct responses to treatment (Cuthbert and Insel, 2013; Decker, 2013). This heterogeneity suggests several etiological and pathophysiological mechanisms related to the same disorder, so that multiple individuals with the same disorder may nevertheless have very different courses of illness and treatment outcomes (Feczko et al., 2019). Thus, reducing the high heterogeneity in psychiatry by better clarifying the biology underlying these disorders may help advance treatments by identifying more precise neurobiological targets and personalized interventions (biotypes) as well as accurate biomarkers for diagnosis, prognosis, and treatment prediction. As one salient example, a study that analyzed data from a randomized, controlled trial of infliximab to treat bipolar depression identified inflammatory biotypes based on peripheral cytokines that could predict efficacy for decreasing anhedonia, a core symptom of depression associated with poorer outcomes (Bonanni et al., 2019; Lee et al., 2021). This personalized approach also includes recent studies with P2X7 modulators in bipolar depression that have been enrolling participants with higher baseline proinflammatory status. In another study, researchers investigated whether polygenic risk scores for MDD could predict response to lithium in individuals with BD, another example of how biomarkers can help guide better treatments (Machado-Vieira, 2018).

Taken together, the preliminary findings on novel therapies emerging from non-canonical pathways are encouraging. Although the targets have been described for a long time, translating these biomarkers into therapeutics has been challenging. All of them, however, may represent potentially valuable therapeutic alternatives for BD, although their long-term safety, tolerability, and efficacy remain unclear, and the available evidence is insufficient to support their current extensive use. Nevertheless, moving forward, they may hold potential as off-label alternatives for symptom relief and a bridge to conventional treatment therapies for more severe disease cases—for instance, as in treatment-resistant BD and specific clinical dimensions, such as anhedonia, fatigue, sleep, and suicidality. Given that early detection and prompt intervention are known to decelerate illness progression and lessen the clinical burden associated with BD (Culpepper, 2014), developing novel therapies with rapid and sustained clinical benefits remains an urgent focus in psychiatry. This is particularly important given that most of the pathways discussed in this study may also be involved in other psychiatric conditions such as MDD or schizophrenia. Developing medications tailored explicitly for BD and its mood states is likely to translate into new, improved personalized therapies and better outcomes for patients.

## Author contributions

RM-V, AC, CZ, IH, and HM contributed equally to the literature search, generation of the figures, writing, and revision of this manuscript. All authors approved the submitted version.

## Funding

Funding for this work was provided in part by the Intramural Research Program at the National Institute of Mental Health, National Institutes of Health (IRP-NIMH-NIH; ZIAMH002927). The work was completed as part of the authors’ official duties as Government employees.

## Conflict of interest

CZ is listed as a co-inventor on a patent for the use of ketamine in major depression and suicidal ideation; as a co-inventor on a patent for the use of (2*R*,6*R*)-hydroxynorketamine, (*S*)-dehydronorketamine, and other stereoisomeric dehydroxylated and hydroxylated metabolites of (*R,S*)-ketamine metabolites in the treatment of depression and neuropathic pain; and as a co-inventor on a patent application for the use of (2*R*,6*R*)-hydroxynorketamine and (2*S*,6*S*)-hydroxynorketamine in the treatment of depression, anxiety, anhedonia, suicidal ideation, and post-traumatic stress disorders. He has assigned his patent rights to the U.S. government but will share a percentage of any royalties that may be received by the government. RM-V has received consulting fees from Eurofarma Pharmaceuticals, Abbott, and BioStrategies group and has a research contract with Boerhinger Ingelheim, Janssen Pharmaceuticals. RM-V has also received speaker fees from Otsuka, EMS, and Cristalia and is a member of the scientific boards of Symbinas Pharmaceuticals and Allergan. RM-V is the PI for the following grants: NIH (R21HD106779 and R21MH129888), Milken Institute (BD-0000000081), and UT Health (BD2 Integrated Network). HM is the former Global Head of Science for Minds at Johnson and Johnson and has no current conflict of interest to disclose.

The remaining authors declare that the research was conducted in the absence of any commercial or financial relationships that could be construed as a potential conflict of interest.

## Publisher’s note

All claims expressed in this article are solely those of the authors and do not necessarily represent those of their affiliated organizations, or those of the publisher, the editors and the reviewers. Any product that may be evaluated in this article, or claim that may be made by its manufacturer, is not guaranteed or endorsed by the publisher.

## Author disclaimer

The views expressed do not necessarily reflect the views of the NIH, the Department of Health and Human Services, or the United States Government.
